# Society Meetings

**Published:** 1917-09

**Authors:** 


### SOCIETY MEETINGS

Brief reports and announcements of meetings of societies of Western New York. and those of general scope, are requested from Secretaries. Copy should be on hand the fifteenth of the month. Full transactions will be published at cost of composition.

**DOCTOR: Is your Society properly represented here? If not, please bring our standing announcement to the attention of the members.**

**The Medical Society of the County of Erie** held a special military meeting June 27, in Alumni Hall of the Medical Dept. of the University of Buffalo. Capt. H. H. Glosser, M. D., showed lantern slides illustrating the experience of the 74th Reg. N. G. N. Y. at the Texas Border and patriotic addresses were made by Dr. T. H. McKee and Lt. Col. A. H. Briggs, M. D.

**The American Medical Editors' Assn.** at its annual meeting in N. Y., June 4 and 5, agreed to co-operate in the work of securing volunteer medical officers and urged on all medical journals the duty of publishing matter, especially with reference to the Reserve Corps. This journal has already begun this work and will continue to do so. We consider it unnecessary to publish the blank form for volunteering but any physician can secure the same on application.

**The Western N. Y. Veterinary Medical Assn.** held its fourth annual meeting in Buffalo, June 29. The clinic was held in the hospital of the building of the Erie County Society for the Prevention of Cruelty to Animals in West Tupper Street. Papers were read by Dr. J. V. Hills of Gowanda; Dr. F. E. McClelland of Buffalo; Dr. Edward Rafter of Hamburg; Dr. A. Crowforth of Lockport, and Dr. F. F. Fehr of Buffalo. At the evening session, these officers were elected: President, Dr. J. L. Wilder of Akron; Vice-President, Dr. W. E. Frint of Batavia; Secretary, Dr. F. F. Fehr of Buffalo; Censors, Dr. Charles E. Gibbs, Dr. E. L. Volgenau, Dr. John Wende, Dr. George R. Chase, Dr. Edward Rafter, Dr. F. E. McClelland. The next meeting will be held in December.

**The University of Toronto Alumni Assn. of Buffalo and Niagara Falls,** organized in Feb., held its June meeting at the University Club, Buffalo, under the presidency of Dr. John D. Bonnar, who gave an account of the general American Alumni Assn. meeting in N. Y. in June. (Report of this and three preceding meetings received too late for publication in August issue).

---

**The Medical Society of the County of Erie** held a special meeting in Alumni Hall, University of Buffalo, July 30. Major Isaac H. Jones, Medical Officers' Reserve Corps, Philadelphia, demonstrated the revolving chair used for equilibration tests for the Aviation corps, described the technic and principles. Moving pictures were included in the demonstration. 30 units for the examination of applicants for the Aviation corps have been or will be established at various points, and it is estimated that 10 or more can be examined daily at each, so that the 100,000 quota can be filled within a year. An unnoticed but really notable detail was the cordial reference by Major Jones to the studies of Viennese specialists. The medical profession serving in this war will not confuse race hatred with efficiency. A petition was circulated and largely signed, demanding the application of the selective draft to the medical profession, between the age limits of 21 and 45. This petition originated with the State Society and is in harmony with the action of various other states and in opposition to the present attitude of the national authorities with regard to the Medical Dept. of the Officers' Reserve Corps.

